# Mechanical asymmetries remain low-to-moderate during 30 min of self-paced treadmill running

**DOI:** 10.3389/fphys.2023.1289172

**Published:** 2023-12-18

**Authors:** Khouloud Mtibaa, Nidhal Zarrouk, Joong Hyun Ryu, Sébastien Racinais, Olivier Girard

**Affiliations:** ^1^ College of Health and Life Sciences, Hamad Bin Khalifa University, Doha, Qatar; ^2^ Education, Motricity, Sports and Health, High Institute of Sport and Physical Education of Sfax, Sfax, Tunisia; ^3^ Sports Science Department, Aspire Academy, Doha, Qatar; ^4^ Research and Scientific Support, Aspetar Orthopaedic and Sports Medicine Hospital, Doha, Qatar; ^5^ Exercise and Sport Science Department, School of Human Sciences, The University of Western Australia, Perth, WA, Australia

**Keywords:** symmetry angle scores, asymmetry, pacing, running mechanics, ground reaction forces

## Abstract

**Introduction:** We characterized the magnitude and range of gait asymmetry during self-paced treadmill running.

**Methods:** On an instrumented treadmill, twelve trained runners (11 males, 1 female) completed a 30-min self-paced run, during which participants were instructed to cover the most distance possible. Ground reaction force recordings at a constant velocity corresponding to 70% of their maximal aerobic velocity (13.3 ± 0.8 km.h^−1^) allowed for the measurement of running kinetics and kinematics, as well as the calculation of spring-mass characteristics at the beginning, middle, and end of the run (minutes 1, 14, and 29, respectively). Group mean asymmetry scores were assessed using the “symmetry angle” (SA) formulae, where scores of 0% and 100% represent perfect symmetry and perfect asymmetry, respectively.

**Results:** There was no time effect on SA scores for any of the 13 biomechanical variables (*p* ≥ 0.128). Mean SA scores were <2.5% for contact time (0.8% ± 0.7%), flight time (1.4% ± 0.6%), step frequency (0.7% ± 0.3%), duty factor (0.7% ± 0.3%), duration of braking (1.3% ± 0.7%) and push-off phases (0.9% ± 0.8%), as well as peak braking (2.3% ± 1.3%) and push-off forces (1.4% ± 0.9%). Mean SA scores were ≥2.5% for peak vertical loading rate (3.1% ± 1.7%), mean vertical loading rate (3.4% ± 2.1%), peak vertical forces (2.9% ± 2.2%), as well as vertical stiffness (5.2% ± 3.5%) and leg stiffness (2.5% ± 1.5%).

**Conclusion:** Throughout a 30-min running time trial, there were consistently low-to-moderate mechanical asymmetries for spatiotemporal variables, kinetics, and spring-mass model characteristics. This suggests that trained runners maintained relatively even strides during the self-paced treadmill run, with lower extremities behaving similarly when controlling for velocity.

## Introduction

Several factors, both intrinsic (e.g., limb length differences) and extrinsic (e.g., injuries or trauma), can account for the presence of a dominant and non-dominant side in an individual’s body (Bishop et al., 2018). It is therefore not uncommon for gait to exhibit asymmetry even in uninjured athletes. When one side of the body bears a disproportionate load, this uneven stride can adversely affect performance, particularly when it exceeds the maximal capacity of the weaker side (Afonso et al., 2022). In extreme cases, unilateral injuries may develop as the weaker side is not able to tolerate a mechanical loading that exceeds its maximal capacity (Furlong and Egginton, 2018). Therefore, measuring the degree of biomechanical asymmetry in the lower limbs during locomotor tasks such as running can be valuable for prescribing interventions aimed at distributing load appropriately to enhance athletic performance and reduce the risk of injury (Bredeweg et al., 2013; Bishop et al., 2018).

Asymmetry is commonly observed in golfers or racket sport players, where the arm holding the club or racket exhibit greater strength, power, and muscular development due to the repetitive and unilateral nature of these sports (Afonso et al., 2022). On the other hand, activities such as cycling or running are inherently more symmetrical, involving more evenly distributed forces and movements between the left and right sides of the body (Carpes et al., 2010). Even experienced coaches may struggle to identify biomechanical imbalances in the running gait pattern of their athletes with the naked eye, as these imbalances are not always obvious. Therefore, evaluating symmetry in running mechanics, typically derived from ground reaction force (GRF) data, necessitates advanced analysis of an individual’s running pattern. Instrumented treadmills are increasingly being employed to collect numerous consecutive steps and monitor inter-limb differences (Brown et al., 2017; Melo et al., 2020).

In recent years, there has been a growing interest in examining changes in mechanical asymmetry in fatigued runners due to its perceived impact on performance and injury (Heil et al., 2020). Researchers have focussed extensively on alterations in lower limb asymmetry during both maximal (Girard et al., 2017; Girard et al., 2023; Van Alsenoy et al., 2023) and constant submaximal intensity running (Brown et al., 2014; Hanley and Tucker, 2018; Gao et al., 2020; Melo et al., 2020; Gao et al., 2022), yielding conflicting findings (Heil et al., 2020). For instance, elite female athletes performing eight 5-s repeated treadmill sprints showed no significant differences in most stride mechanical variables (Girard et al., 2023). Conversely, amateur runners completing a fixed-pace 10 km run exhibited increased asymmetry and decreased mechanical efficiency (Melo et al., 2020). However the use of pre-determined, fixed physiological intensities in these studies lacks ecological validity, as running velocity is ultimately self-regulated by the individual during exercise.

Recent attention has turned toward examining the biomechanical effects of fatigue during self-paced exercise, where treadmill velocity is freely adjustable, providing a more natural control of stride mechanical patterns (Apte et al., 2021). Surprisingly, modifications in asymmetry in response to self-paced exercise have not been thoroughly documented (Heil et al., 2020). During perceptually regulated interval runs (4 × 4-min intervals; 3-min passive recovery) on an instrumented treadmill, bilateral leg differences remained consistent both between and within intervals (Girard et al., 2021). Most previous research on asymmetry modifications has primarily focused on adjustments occurring between the onset and end of the run (Ryu, 2018) or assessed pre-post differences (Brown et al., 2014; Radzak et al., 2017; Gao et al., 2020; Gao et al., 2022), sometimes using nonspecific procedures (i.e., unilateral jump tests; Bishop et al., 2021) that do not reflect actual running demands. Additionally, previous assessments of self-paced runs have often been limited to spatiotemporal (i.e., contact time; Ammann et al., 2016) or vertical GRF variables (Jacques et al., 2021). This is problematic since, during acute intense fatigue, peak braking forces, as opposed to push-off forces, exhibit four times larger deviations from symmetry in the anteroposterior direction (Girard et al., 2023).

The aim of this study was to analyse stride mechanical asymmetries, including phase duration, peak forces, and impulses, during a 30-min self-paced treadmill run at maximum effort. We hypothesized that running mechanics would exhibit varying degrees of inter-limb differences depending on the variable of interest, with greater differences observed for horizontally-derived variables compared to vertically-derived ones, and that these differences would be magnified as the distance covered increased. Asymmetry evaluation, often reliant on a restricted number of parameters focusing on vertical GRF (i.e., neglecting insights into horizontal asymmetries during braking and push-off; Afonso et al., 2022) and/or using non-specific tasks (i.e., unilateral jumps; Bishop et al., 2021), falls short of capturing the complexity of fatigue-related changes. By examining the time course of adjustments to running mechanics during the actual running protocol, the significance of this study includes shedding light on the role of metric-dependent factors in fatigue-related asymmetry progression during self-paced treadmill running.

## Methods

### Participants

Out of the initial convenience sample of 18 participants, four did not meet the selection criteria of a maximal aerobic velocity ≥18 km.h^−1^, and two dropped out for medical reasons. Twelve participants (11 males, 1 female; age: 33.7 ± 5.6 yrs; height: 1.77 ± 0.06 m; body mass: 74.3 ± 6.8 kg) were recruited to participate in the study. All participants self-reported to be healthy, not under current medication and free of any lower-limb injury for at least 2 years before the study. The study was approved by the local Ethics committee and conducted in accordance with the Declaration of Helsinki, with written informed consent obtained from participants.

### Procedures

While this study was conducted as part of a larger project investigating the impact of heat stress on ankle proprioception and running gait patterns (Mtibaa et al., 2019), the primary outcome measures in this study (gait asymmetries) are distinct from those analysed previously.

Approximately 1 week before testing, participants completed a pre-experimental session. First, they performed an incremental test consisting of four 4-min stages (8.5 km.h^−1^, 10 km.h^−1^, 11.5 km.h^−1^ and 13 km.h^−1^), followed by increments of 1 km.h^−1^ every minute until exhaustion to estimate their maximal aerobic velocity (average: 19.0 ± 1.1 km.h^−1^, range: 18.0–21.3 km.h^−1^). Subsequently, they engaged in a 10-min self-paced run for habituation.

The main visit involved a 30-min self-paced treadmill run. After a 10-min standardized warm-up, participants rested for 5 min (quiet standing) before starting the 30-min self-paced run. Minutes 1, 14, and 29 of the run (Start, Mid, End, respectively) were performed at an externally-imposed velocity corresponding to 70% of their maximal aerobic velocity (average: 13.0 ± 0.8 km h^−1^, range: 12.6–14.9 km.h^−1^), ensuring that running mechanics could be compared at a consistent velocity. During the main part of the run, participants were instructed to cover the greatest distance possible. A timer-screen was positioned in front of the runners to continuously display the running time, while participants remained unaware of the actual treadmill velocities. They were free to make decisions regarding whether and how treadmill velocity needed adjustment. The same experimenter controlled the treadmill, which was initially set to the specified imposed velocity for each participant before the test began and could be adjusted with a precision of 0.1 km.h^−1^ at any time upon the participant’s request. Participants wore their own habitual shoes.

Participants completed their run on an instrumented treadmill (ADAL3D-WR, Medical Development–HEF Tecmachine, Andrezieux-Boutheon, France) located within an indoor facility maintained at 22°C and 48% relative humidity. The treadmill was mounted on a highly rigid metal frame fixed to the ground through four piezoelectric force transducers (KI 9077b; Kistler, Winterthur, Switzerland) and installed on a specially engineered concrete slab to ensure maximal rigidity of the supporting ground.

### Running mechanics

Ten consecutive steps (comprising five right and five left leg foot contacts, averaged separately for each side) that commenced at the 15th second of each 1-min constant-velocity running bout were subjected to analysis (i.e., after running for ∼15 s, ∼14 min 15 s, and ∼19 min 15 s, respectively). This procedure for quantifying gait asymmetry has been consistently applied in previous analyses (Bredeweg et al., 2013; Girard et al., 2019; Girard et al., 2021). Importantly, the treadmill velocity was kept undisclosed from the participants, who were instructed to “run normally” throughout each run without knowledge of the exact moment of sampling (Morin et al., 2009). The experimenter could discreetly trigger a 10-s data sampling by clicking a mouse button placed outside the participant’s field of vision.

Running mechanics were continuously sampled at a rate of 1,000 Hz. Subsequently, after appropriate filtering (Butterworth-type, 30 Hz low-pass filter), instantaneous data for vertical, horizontal and total (resultant) GRF were averaged for each support phase when the vertical force exceeded 30 N. Contact time (s), flight time (s), step frequency (Hz) and duty factor (contact time relative to total stride time) were calculated. Foot strike and toe-off instants were determined as the moments were the vertical GRF crossed above and below 30 N, respectively. Step frequency was determined as the inverse of step duration, which was defined as the time from the foot strike of one leg to the next foot strike of the other leg. Contact time represented the duration from foot strike to toe-off, while flight time was the duration from toe-off to foot strike. Durations of the braking and push-off phases (s) were calculated, as were the peak braking and peak push-off forces (BW). Finally, both peak and mean vertical loading rates over the initial 50 m (LR) were computed as the peak or mean values of the time derivate of the vertical force signal within the first 50 m of the support phase and expressed in BW/s (Girard et al., 2023).

A linear spring-mass model was used to characterize the mechanical lower limb behavior of the lower limbs (for details, see McMahon and Cheng, 1990). Vertical stiffness (kN.m^−1^) was calculated as the ratio of peak vertical forces (Fz_max_ in N) to the maximal vertical downward displacement of centre of mass (∆z in m), which was determined by double integration of vertical acceleration of centre of mass over time during ground contact. Leg stiffness (kN.m^−1^) was calculated as the ratio of peak vertical forces to the maximum leg spring compression (∆L in m) [∆z + L_0_ − √L_0_^2^ − (0.5 × running velocity × contact time)^2^], both occurring at mid-stance. Initial leg length (L_0_, great trochanter to ground distance in a standing position) was determined from participant’s stature as L_0_ = 0.53 × stature (Morin et al., 2005).

### Symmetry angle

To assess inter-leg symmetry for each participant, the symmetry angle (SA) equation, as described by Zifchock et al. (2008), was employed:

Symmetry angle (SA) =

$$\frac{\left|\;45{}^{\circ}\;-\;\left(\;\tan^{-1}\;\left[\;\frac{left}{right}\;\right]\;\right)\;\right|}{90{}^{\circ}}\times 100$$

but if

$$\left(45{}^{\circ}-\tan^{-1}\left[\frac{left}{right}\right]\right)>90{}^{\circ}$$

then

$$\frac{\left|\;45{}^{\circ}\;-\;\left(\;\tan^{-1}\;\left[\;\frac{left}{right}\;\right]-180{}^{\circ}\right)\;\right|}{90{}^{\circ}}\times 100$$

The SA is calculated as the arctan function of the ratio between two values from each leg, with a SA score of 0% signifying perfect symmetry and 100% indicating perfect asymmetry.

### Statistical analysis

Values are presented as mean ± SD and 95% confidence interval (CI_95%_). For the 13 biomechanical variables, the effect of time was determined by a single factor ANOVA for repeated measures across each time point (Start, Mid and End). To assess assumptions of variance, Mauchly’s test of sphericity was performed using all ANOVA results. A Bonferroni post-hoc multiple comparison was performed if a significant main effect was observed. For each ANOVA, was calculated as measures of Effect sizes were described in terms of partial eta-squared (η^2^, with η^2^ ≥ 0.06 representing a *moderate* effect and η^2^ ≥ 0.14 a *large* effect). All statistical calculations were performed using SPSS statistical software V.27.0 (IBM Corp., Armonk, NY, United States). The significance level was set at *p* < 0.05.

## Results

**FIGURE 1:**
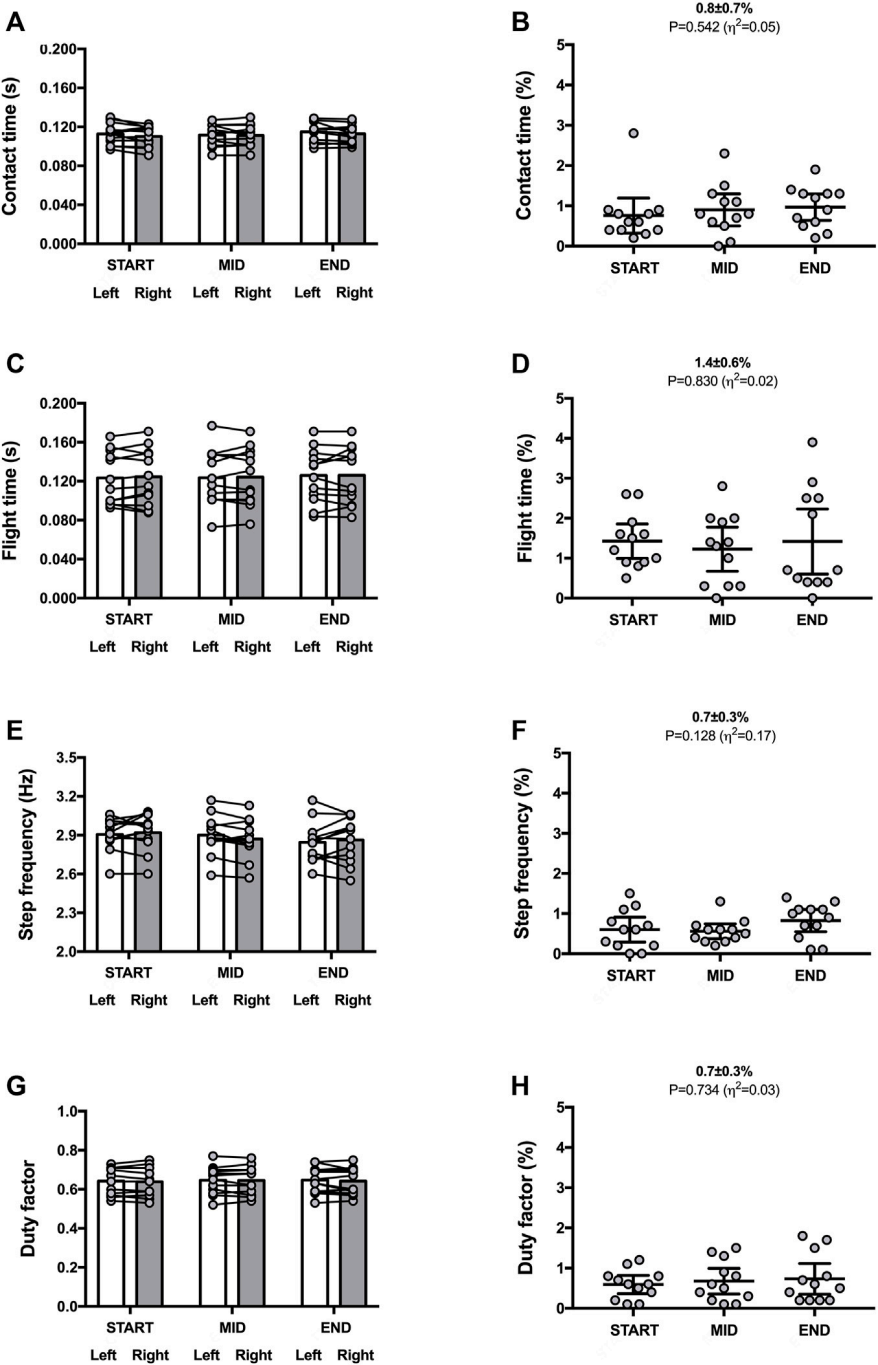
Spatio-temporal variables for both legs (left panels) and symmetry angle scores (right panels) during the 30-min time trial. Contact time **(A,B)**; flight time **(C,D)**; step frequency **(E,F)**; duty factor **(G,H)**. Values are mean with 95% confidence interval (*n* = 12). Symmetry angle score of 0% indicates perfect symmetry and 100% indicates perfect asymmetry.

**FIGURE 2:**
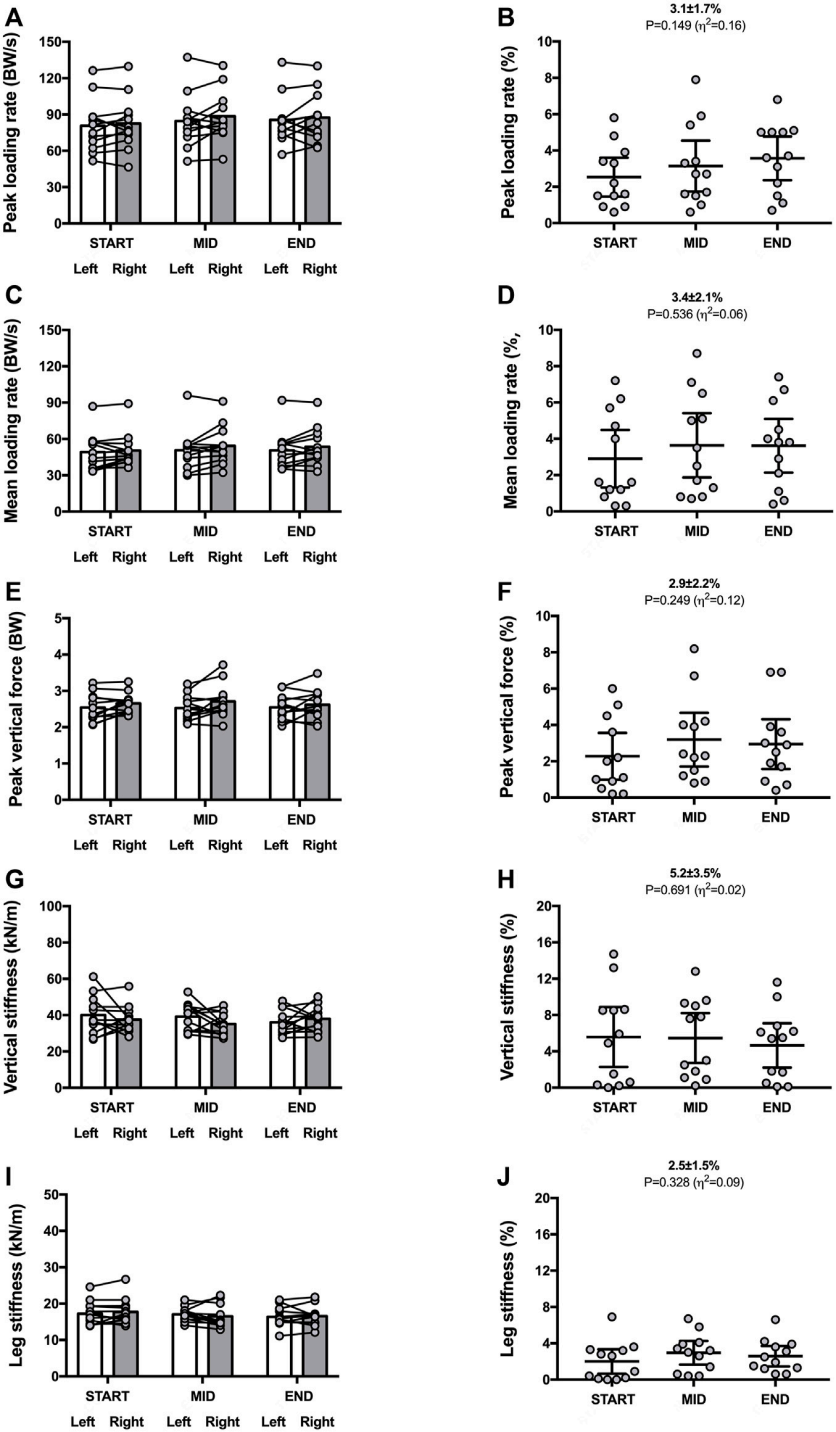
Vertical force-related variables and sprint mass characteristics for both legs (left panels) and symmetry angle scores (right panels) during the 30-min time trial. Peak loading rate **(A,B)**; mean loading rate **(C,D)**; peak vertical force **(E,F)**; vertical stiffness **(G,H)**; leg stiffness **(I,J)**. Values are mean with 95% confidence interval (*n* = 12). Symmetry angle score of 0% indicates perfect symmetry and 100% indicates perfect asymmetry.

**FIGURE 3:**
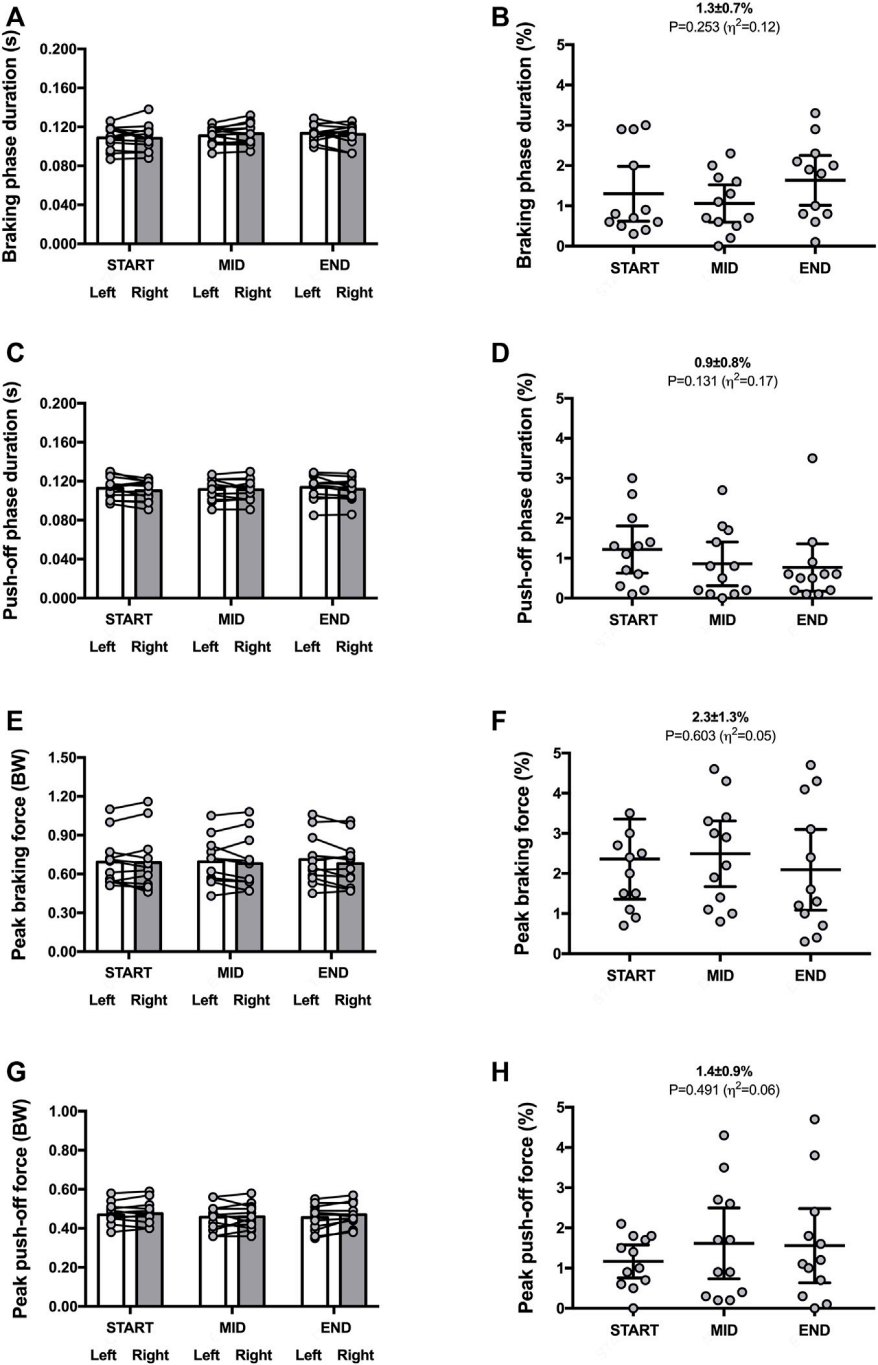
Horizontal force-related variables for both legs (left panels) and symmetry angle scores (right panels) during the 30-min time trial. Braking phase duration **(A,B)**; push-off phase duration **(C,D)**; peak braking force **(E,F)**; peak push-off force **(G,H)**. Values are mean with 95% confidence interval (*n* = 12). Symmetry angle score of 0% indicates perfect symmetry and 100% indicates perfect asymmetry.

There was no influence of time on SA scores for any of the 13 biomechanical variables (*p* ≥ 0.128; Figures 1–3). Consequently, group means and the range of SA scores are presented below in the text as pooled values, corresponding to the average across the three time points. This averaging procedure offers material benchmark for expected levels of asymmetry during a 30-min time trial for each metric.

Mean SA scores were ∼1% for contact time [0.8% ± 0.7% (CI_95%_ 0.4–1.3); range: 0.1–2.9], flight time [1.4% ± 0.6% (CI_95%_ 0.9–1.8); range: 0.6–2.8], step frequency [0.7% ± 0.3% (CI_95%_ 0.5–0.9); range: 0.2–1.0], and duty factor [0.7% ± 0.3% (CI_95%_ 0.4–0.9); range: 0.2–1.4] (Figure 1).

Mean SA scores were ∼3% for peak loading rate [3.1% ± 1.7% (CI_95%_ 1.9–3.4); range: 1.1–5.6], mean loading rate [3.4% ± 2.1% (CI_95%_ 2.1–4.7); range: 1.0–6.2] and peak vertical forces [2.9% ± 2.2% (CI_95%_ 1.6–4.0); range: 0.4–6.9] (Figure 2).

Vertical stiffness and leg stiffness presented mean SA values of 5.2% ± 3.5% [(CI_95%_ 3.0–7.5); range: 3.0–10.1] and 2.5% ± 1.5% [(CI_95%_ 1.6–3.5); range: 0.4–4.4], respectively (Figure 2).

Mean SA scores were ∼1–2.5% for duration of braking [1.3% ± 0.7% (CI_95%_ 0.9–1.7); range: 0.2–2.4] and push-off phases [0.9% ± 0.8% (CI_95%_ 0.4–1.5); range: 0.1–3.1] as well as peak braking [2.3% ± 1.3% (CI_95%_ 1.5–3.1); range: 0.9–5.3] and push-off forces [1.4% ± 0.9% (CI_95%_ 0.9–2.0); range: 0.2–3.3] (Figure 3).

The average running velocity was 14.7 ± 1.7 km.h^−1^, resulting in a total distance covered of 7,276 ± 703 m. Running velocity did not differ between the first and second half of the run (14.3 ± 1.5 vs. 15.1 ± 1.9 km.h^−1^; *p* = 0.360).

## Discussion

### Summary of main findings

Contrary to our hypothesis, there were no modifications in gait asymmetries, as measured at the beginning, middle, and end of a 30-min time trial in trained runners. Overall, low-to-moderate mechanical asymmetries persisted for spatio-temporal variables, kinetics and spring-mass model characteristics. This occurred despite exercise-induced changes in stride (i.e., increase in step length and decrease in step frequency) and angular (i.e., upswing of the pelvis and a decrease in ankle dorsi-flexion angle) kinematics, which were previously described elsewhere (Mtibaa et al., 2019). Overall, a 30-min self-paced treadmill run did not subject one side of the body to greater mechanical constraints than the other.

### Constant asymmetry throughout the run

Previous running studies assessing the biomechanical manifestation of fatigue on side-to-side differences have yield mixed findings, with some reporting unchanged asymmetries (Brown et al., 2014; Ammann et al., 2016; Hanley and Tucker, 2018) while other indicate increased ones (Radzak et al., 2017; Gao et al., 2020; Gao et al., 2022). These inconsistent results may be attributed to the varying levels of fatigue experienced by runners, which likely affect the load on the neuromuscular system. Factors contributing to this variability include exercise variables (i.e., selected running velocity and/or duration; Mo et al., 2020) and properties of the running surface (treadmill vs. track; Robadey et al., 2018). Additionally, individual factors such as the athletes’ training background (i.e., gender, weekly mileage; Mo et al., 2020) and unique running styles (i.e., foot strike pattern was not assessed here; Larson et al., 2011) could also play a role. Similar to the consistently low SA scores reported throughout a 30-min self-paced treadmill run, Ammann et al. (2016) did not find substantial differences in asymmetry throughout (i.e., every 200 m) a 5,000 m overground time trial. Assessing changes in bilateral leg asymmetry during self-paced runs, as proposed by Ammann et al. (2016), can present methodological challenges when distinguishing genuine fatigue effects from auto-regulatory mechanisms linked to pacing strategies. To overcome this limitation, we externally imposed treadmill velocity for 1-min intervals at the beginning, middle, and end of the run (i.e., despite participants being free to adjust it during the rest of the run) to explore potential changes in SA scores.

### SA scores are metric-dependent

The mean SA scores for time-based gait parameters (e.g., contact and flight times, step frequency and duty factor, braking and push-off phase durations) during the run consistently remained low, generally below 1.5%. This pattern aligns with findings from other studies (Girard et al., 2023; Van Alsenoy et al., 2023), suggesting that, within this athletic cohort and under the current conditions, spatio-temporal gait variables exhibit a high degree of symmetry and remain stable over a 30-min run. Contrastingly, the highest SA scores were observed for vertical stiffness, slightly exceeding 5%. We previously reported similar findings, indicating that vertical stiffness (and spring-mass characteristics in general) is the biomechanical variable that shows the largest differences between the two legs across a range of constant velocities (Girard et al., 2019). While these observations reinforce that the magnitude of side-to-side differences depends on the specific metric being examined, we add the novel observation that the range of mean SA scores for vertically-derived measures (ranging from 2% to 5%) was more consistent compared to the mechanical variables derived from the horizontal GRF signal. This differs from what is typically observed during single (Brown et al., 2017) or repeated treadmill sprints (Girard et al., 2017; Girard et al., 2023) where variables derived from the horizontal GRF signal tend to be the most asymmetrical. Contrary to our hypothesis, adjustments occurring during the braking and push-off phases exhibited comparable SA scores. This finding is consistent with previous observations during repeated-running sprints (Van Alsenoy et al., 2023) and perceptually-regulated interval running (Girard et al., 2021). This indicates that alterations in foot strike pattern throughout the run were not significantly amplified on either the weaker or strong leg by braking more in the early stance phase and pushing less forcefully forward. It is important to exercise caution in utilizing and interpreting group analyses where asymmetry is the main outcome, as the magnitude of between-limb differences during a self-paced treadmill run varies depending on the specific metric being assessed.

### Individual responses

Consistent with previous running studies (Afonso et al., 2022), there was notable inter-individual variability across all metrics. These varying degrees of mechanical asymmetry stem from functional disparities between participants in terms of how each leg contributes to neuromuscular control during the stance phase (Read et al., 2019). Because surface EMG (i.e., muscle activation patterns; Jacques et al., 2021), wearables (3D trunk accelerometry parameters; Schütte et al., 2015), and high-speed cameras (i.e., joint angles at touch-down/take off; Gao et al., 2020; Bissas et al., 2022) data from dominant and non-dominant legs were not captured, we can not rule out the possibility that neuromuscular compensatory strategies may have emerged to maintain consistent asymmetry levels throughout the run. Regardless, the range of SA scores for most runners was contained within approximately twice the magnitude of the mean value, irrespective of the time interval. This pattern mirrors observations made during treadmill graded exercise test (Girard et al., 2022) and at submaximal constant-velocity (Hanley and Tucker, 2018) or repeated sprints (Van Alsenoy et al., 2023). For instance, Hanley and Tucker (2018) found substantial inter-individual differences on an individual level, especially for spatiotemporal parameters, during a 10,000 m treadmill run at constant velocity. They considered the subjects’ inter-limb difference to be asymmetrical if more than half of their SA values exceeded a certain threshold.

The lack of significant group mean kinetic and kinematic inter-limb differences indicates no discernible differences in the load experienced by each side of the lower extremity joints during the course of the run. However, for a given participant, we could not confirm whether our running protocol exposed one side of the body to more mechanical stress than the other at specific time or distance intervals. It is reasonable to assume that participants adopting different pacing strategies might have experienced varying patterns of mechanical adaptations throughout the 30-min run. To explore this further, it would be beneficial to assess the consistency of the direction of asymmetry (i.e., which limb is favored; Afonso et al., 2022) at more frequent time intervals, such as every 5 min. This approach could shed light on whether, for some athletes, the limb producing the greatest values occasionally switched sides during the run.

### Limitations and additional considerations

This study is not without limitations. Firstly, our experienced runners may have benefited from the moving belt on the instrumented treadmill, which could potentially mask exercise-induced gait asymmetries. This contrasts with overground runs, where foot strikes on solid ground are monitored by a series of force plates. In support of this notion, Robadey et al. (2018) reported higher gait asymmetry during overground running, especially in individuals with laterally pronounced knee osteoarthritis, compared to treadmill running. Arguably, running velocity tends to be more variable during overground, even when conditions are carefully regulated, which might contribute to increased asymmetry (Riley et al., 2008). Secondly, our study did not control for the use of specific foam (carbon plate) shoes or foot orthoses by runners who may have worn them for comfort or to mitigate gait alterations while wearing their habitual footwear. However, it is important to mention that Van Alsenoy et al. (2023) found that wearing custom foot orthoses did not significantly alter the observed low-to-moderate natural stride mechanical asymmetries in well-trained runners experiencing intense fatigue. Thirdly, we did not assess laterality or leg preference in our study, which could have provided insights into whether limb dominance influenced mechanical side-to-side differences during treadmill runs (Brown et al., 2014). Finally, emerging statistical approaches that quantify fatigue-related changes in mechanical asymmetry, such as statistical parametric mapping that expresses GRF data as a function of normalized stance phase duration rather than discrete values (i.e., peak braking or push-off forces), could be beneficial in future studies (Jacques et al., 2021).

## Conclusion

In trained runners, there was no noticeable difference in asymmetries for vertically- and horizontally-derived parameters during 30-min self-paced track running. Irrespective of time interval, bilateral leg differences during the braking and push-off phases were generally low-to-moderate, and comparable to vertical GRF asymmetry. These findings have clear methodological implications when assessing mechanical alterations during self-paced runs. Experimental procedures for characterizing fatigue-related mechanical changes in self-paced treadmill runs could be simplified in uninjured, trained runners by collecting leg mechanical data from only one side.

## Data Availability

The raw data supporting the conclusion of this article will be made available by the authors, without undue reservation.
